# Case Report: A case of rituximab for membranous nephropathy secondary to primary Sjogren’s syndrome and literature review

**DOI:** 10.3389/fimmu.2026.1682660

**Published:** 2026-03-31

**Authors:** XiaoJuan Fu, Fang Gao, Li Zang, Nan Mao

**Affiliations:** 1 Department of Nephrology, the First Affiliated Hospital of Chengdu Medical College, Chengdu, China; 2 School of Clinical Medicine College, Chengdu Medical College, Chengdu, China

**Keywords:** membranous nephropathy, nephronic syndrome, primary Sjögren’s syndrome, rituximab, secondary glomerulonephritis

## Abstract

**Background:**

Primary Sjögren’s syndrome (pSS) is a chronic autoimmune disease characterized by lymphoplasmacytic infiltration of exocrine glands, leading to dry eyes and mouth. Renal involvement, particularly glomerulonephritis, occurs in approximately 5% of pSS patients. Membranous nephropathy (MN) is one of the less common forms of renal involvement in pSS.

**Case presentation:**

A 48-year-old female with a history of oral and ocular dryness for several years and bilateral symmetrical lower extremity edema for three months was admitted to our hospital. Laboratory findings revealed moderate anemia, hypoalbuminemia, and nephrotic-range proteinuria. Schirmer’s test, tear break-up time, and lip biopsy confirmed the diagnosis of pSS. Renal biopsy demonstrated widespread mesangial hypercellularity and mesangial matrix expansion, thickening of the glomerular basement membrane(GBM) with prominent spikes, and subepithelial, intramembranous and mesangial electron-dense deposits under electron microscopy. Immunofluorescence showed diffuse fine granular deposits of IgG (predominantly IgG1 and IgG2). Based on these findings, secondary membranous nephropathy due to pSS was diagnosed.

**Treatment and outcome:**

The patient was treated with prednisone (30 mg/day) and two infusions of rituximab (1 g each, administered 2 weeks apart). After treatment, her 24-hour urinary protein level decreased from 8.5 g to 2.2 g. The dose of glucocorticoids was gradually tapered off. During follow-up, an additional 1 g of rituximab was administered six months later, resulting in a further reduction of proteinuria to 1.3 g/24 hours.

**Conclusion:**

This case highlights the effectiveness of rituximab in managing secondary membranous nephropathy associated with pSS. Further studies are needed to clarify the underlying mechanisms and optimize treatment strategies for this condition.

## Introduction

Primary Sjögren’s syndrome (pSS) is a progressive autoimmune disease characterized by lymphoplasmacytic cell infiltration of the exocrine glands, chiefly the lacrimal and salivary glands (1), leading to xerophthalmia and xerostomia. While, the inflammatory process seems to be extended to the exocrine glands, including but not limited to lung, liver, kidney, skin, nerve, blood and so on (2). In accordance with the underlying pathology, extraglandular manifestations are related to either periepithelial infiltration of parenchymal organs like kidney, liver and lung or resulting from immunocomplex deposition on account of B cell hyperactivity such as glomerulonephritis, peripheral neuropathy and purpura (3–5). Renal involvement, one of the most frequently extraglandular manifestations, occurs roughly in 5% patients with primary Sjögren’s syndrome (6), presenting in two forms: the relatively more common is tubulointerstitial nephritis(TIN) attributed to lymphocytic infiltration while less common is immune complex related glomerulonephritis. Those have been reported usual glomerular lesions including mesangial proliferative glomerulonephritis/IgA nephropathy (7), focal segmental glomerulosclerosis (FSGS) (8), membrano-proliferative glomerulonephritis (7, 8), minimal change disease (8, 9) and membranous nephropathy (7, 8).

Currently, symptomatic treatment remains the primary approach for managing primary Sjögren’s syndrome. Clinicians may choose appropriate glucocorticoid and/or immunosuppressive agents based on disease activity and affected systems by virtue of experience. However, a comprehensive treatment that effectively addresses both glandular symptoms and systemic organ complications is yet not to be established. In view of the pronounced hyperactivity of B cells, B cell depletion and/or suppression of B signals related to their differentiation and survival seems to provide a reasonable therapeutic approach with positive outcomes (10, 11). Rituximab is such a human/murine chimeric monoclonal antibody that specifically targeted against CD20 antigen resulting in B cell depletion, which has a advantage in improving glandular function, controlling systemic inflammation, and reducing reliance on corticosteroids (12).

Here we reported a case of a woman diagnosed with membranous nephropathy secondary to Sjögren’s syndrome, and after treatment with corticosteroids together with rituximab, xerophthalmia, xerostomia as well as proteinuria were relieved.

## Case presentation

Informed consent was obtained from the patient before the case published. A forty-eight-year-old female suffered from oral and ocular dryness with uncertain time at least for a few years and bilateral symmetrical pitting edema of the lower extremities for the past three months was admitted to our hospital in May 2022, with no visible signs of macroscopic hematuria, urinary frequency, oliguria and nocturia. She had no fever, headache, abdominal pain, diffuse arthralgias, aversion to oily food, coughing, sputum production, chest pain or hemoptysis, alopecias, photoallergy, oral ulcerations, butterfly erythema, as well as weight loss. More than a decade back, she had a history of neuromyelitis optica spectrum disorder, leaving paralysis and blindness. She had no familial or genetic background of renal disease. Physical examination manifested severely pretibial pitting edema and muscle weakness and dental caries. She was overweight that the Body Mass Index(BMI) was 31.2kg/m^2^. She had no parotid enlargement, arthritis, rash and lymphadenopathy. Laboratory findings revealed moderate anemia, hypoalbuminemia and nephrotic-range proteinuria. Detailed laboratory results were listed in **Table 1**.

**Table 1:** Main laboratory data of patient on admission.

Blood analysis					
WBC	8040	/*μ*L	BUN	21.2	mg/dL
RBC	2920000	/*μ*L	Cr	0.58	mg/dL
Hb	76	g/L	eGFR 117.3 ml/min.1.73m^2^		
Plt	359000	/*μ*L	UA	5.4	mg/dL
TP	5.3	g/dL	Na	142.1	mmol/L
ALB	1.7	g/dL	K	4.35	mmol/L
GLB	2.6	g/dL	Ca	2.02	mmol/L
TG	286.7	mg/dL	Cl	112.8	mmol/L
CHOL	238.9	mg/dL	Mg	0.83	mmol/L
LDL-C	134.9	mg/dL	P	1.13	mmol/L
AST	14	U/L	CRP	0.03	mg/dL
ALT	10	U/L	ESR	62	mm/h
ALP	70	U/L	Ferritin	11.8	ng/mL
Immune-related outcome					
C3	84	mg/dL	pANCA	1:20	
C4	14	mg/dL	cANCA	(—)	
IgA	334	mg/dL	MPO	(—)	
IgG	356	mg/dL	PR3	(—)	
IgM	19.4	mg/dL	GBM	(—)	
Cryoglobulins	(—)		Anti-PLA2R	(—)	
Serum immunofixation M protein (—)			Anti-THSD7A	(—)	
Light chain κ	320	mg/dL	ANA	1:100	
Light chain λ	277	mg/dL	dsDNA	(—)	
κ/λ	1.16		Anti-SS-A/Ro antibody	(++)	
HBV	(—)		Anti-SS-B/La antibody	(—)	
HCV	(—)		Anti-Scl-70 antibody	(—)	
HIV	(—)		Anti-Jo-1 antibody	(—)	
Anti-nucleosome antibody	(—)		Anti-histone antibody	(—)	
Anti-Smith antibody	(—)		Anti-U1-snRNP	(—)	
Anti-ribosomal P	(—)		Anti-PCNA	(—)	
Tumor markers					
CA199	(—)		CA125	(—)	
CA72-4	(—)		AFP	(—)	
CEA	(—)		NSE	(—)	
Cyfra21-1	(—)		T-PSA	(—)	
F-PSA	(—)		Pro-GRP	(—)	
PG-I	(—)		PG-II	(—)	
PG-I/PG-II	(—)				
Urinalysis					
pH	7.5		WBC/HP	1-4	
UP	3+		RBC/HP	0	
24-TP	8.5g		Cast/HP	0	

WBC, White Blood Cell; RBC, Red Blood Cell; Hb, Hemoglobin; Plt, Platelet; TP, Total Protein; ALB, Albumin; GLB, Globulin; TG, Triglyceride; CHOL, Cholesterol; LDL-C, Low Density Lipoprotein Cholesterol; AST, Aspertate Aminotransferase; ALT, Alanine Aminotransferase; ALP, Alkaline phosphatase; BUN, Blood Urea Nitrogen; Cr, Creatinine; eGFR, Estimated Glomerular Filtration Rate; UA, Uric Acid; Na, Sodium; K, Potassium; Cl, Chloride; Ca, Calcium; P, Phosphorus; CRP, C-reactive Protein; ESR, Erythrocyte Sedimentation Rate; C3, Complement 3; C4, Complement 4; IgA, Immunoglobulin A; IgG, Immunoglobulin G; IgM, Immunoglobulin M; HBV, Hepatitis B Virus; HCV, Hepatitis C Virus; HIV, Human Immunodeficiency Virus; ANCA, Antineutrophil Cytoplasmic Antibody; MPO, Myeloperoxidase; PR3, Proteinase 3; GBM, Glomerular Basement Membrane; PLA2R, Phospholipase A2 receptor antibody; THSD7A, Thrombospondin Type-1 Domain-Containing 7A;ANA, Anti-nuclear Antibody; dsDNA, Double-stranded Deoxyribonucleic Acid; SS, Sjögren’s Syndrome; Scl, Scleroderma; PCNA, Proliferating Cell Nuclear Antigen; CENP-B, Centromere B Protein; CA199, Carbohydate Antigen 199; CA125, Carbohydate Antigen 125; CA72-4, Carbohydate Antigen 72-4; AFP, Alpha-fetoprotein; CEA, Carcino-embryonic Antigen; NSE, Neuron Specific Enolase; Cyfra21-1, Cytokeratin 19 Fragment; PSA, Prostate Specific Antigen; Pro-GRP, Pro-Gastrin-Releasing Peptide; PG, Pepsinogen.UP, Urinary Protein.

**Figure 1:**
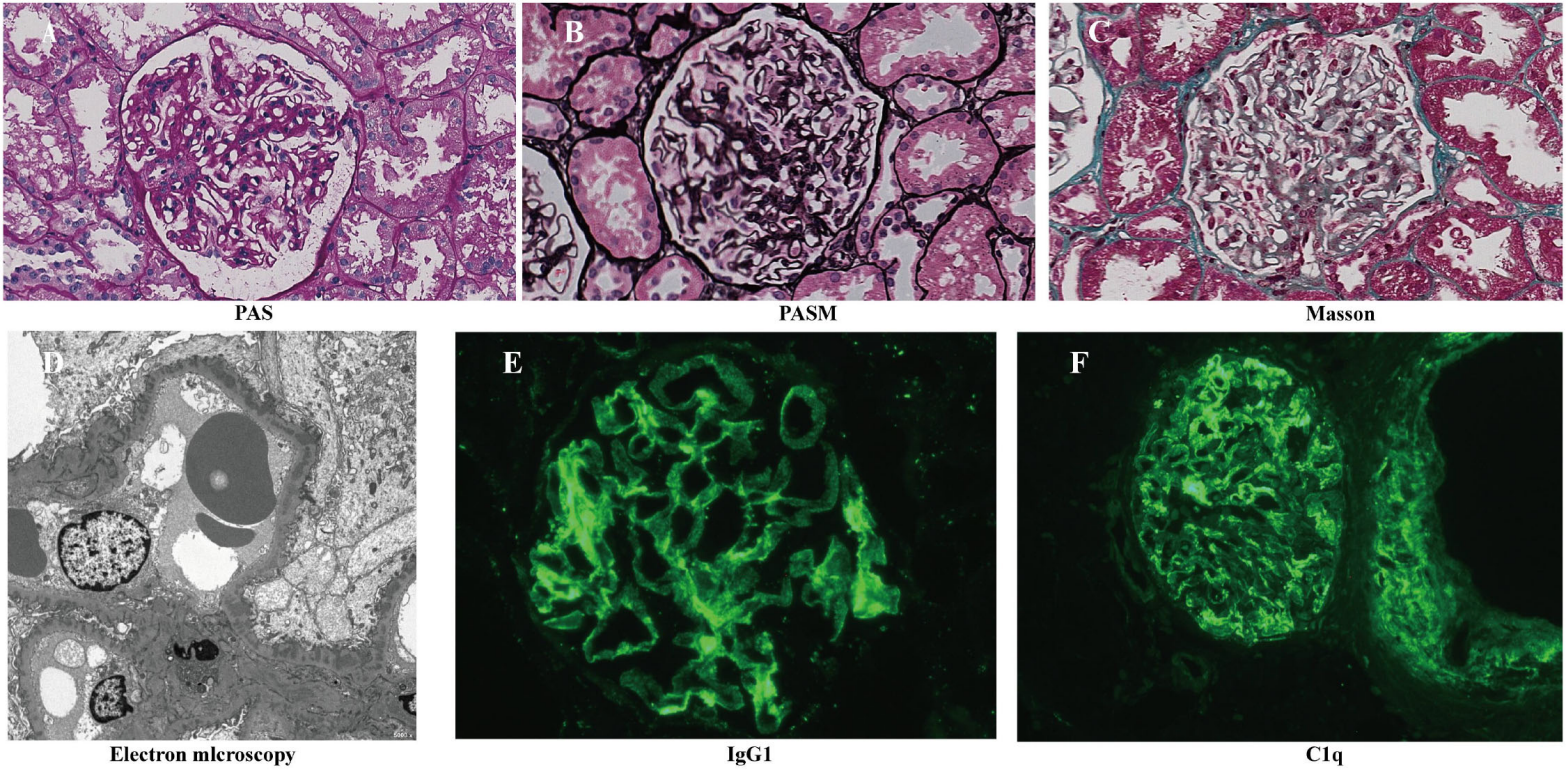
Kidney biopsy findings: **(A)** Widespread mesangial hypercellularity and mesangial matrix expansion(PAS staining, 400x). **(B)** Shown are thickening of glomerular basement membrane where prominent spikes can be observed(PASM staining, 400x). **(C)** Shown are fuchsinophilic protein deposited in subepithelial and mesangial areas(Masson’s trichrome staining, 400x). **(D)** Electron-dense deposites are observed in subepithelial, intramembrane and mesangial regions(Electron microscope, 5000x). **(E)** Immunofluorescence shows a strong positive reaction of IgG1. **(F)** Immunofluorescence shows a strong positive reaction of IgG1.

For the sake of determining the renal pathology, she received an invasive operation, namely percutaneous selective renal biopsy. Under light microscopy, the kidney biopsy contained a total of ten glomeruli, with no visible global and segmental glomerulosclerosis. All glomeruli showed widespread mesangial hypercellularity and mesangial matrix expansion with thickening of glomerular basement membrane where prominent spikes could be observed. Abundant fuchsinophilic protein deposited in subepithelial and mesangial areas. Neither fibrinoid necrosis, wire loop lesion nor crescent formation was detected. The renal tubular epithelial cells had the changes of vacuolar and granular degeneration with few tubular lumens dilatation, epithelial exfoliation, loss of brush border as well as several tubule atrophy, nevertheless without inflammatory cells infiltration in the interstitial tissue. Under electron microscope, the basement membrane was irregularly thickened to a thickness of 1200nm and a large amount of electron-dense deposites were observed in subepithelial, intramembranous and mesangial regions. Immunofluorescence showed diffuse and globular fine granular deposits of IgG +++(especially IgG1 +++, IgG2 ++, IgG4—), along the capillary loops and mesangial areas, while the presence of anti-PLA2R antibodies and anti-THSD7A in kidney tissue are not been detected. Overall consideration, the diagnosis of membranous nephropathy secondary to Sjogren’s Syndrome was made (**Figure 1**).

In view of anemia together with the anomaly ratio of κ/λ, low level of ferritin and transferrin saturation was detected. Morphological examination of bone marrow cells suggested that mature red blood cells varied in size, with an enlarged central pale staining area, and target-shaped red blood cells could be seen. Considering of oral and ocular dryness together with anti-SSA/Ro antibody positivity, we had carried out a series of examinations that an unstimulated salivary flow rate<0.1ml/minute, tear break-up time<10seconds and lip biopsy presented focal lymphocytic sialadenitis with number of foci >50 lymphocytes per 4 mm^2^ of tissue. Tumor-associated membranous nephropathy could also present with IgG1/IgG2 dominance. Therefore, it is necessary to screen for tumors. We conducted additional chest and abdominal CT scans for the patient, which did not reveal any tumor lesions. At the same time, we completed the tests for tumor markers and the results were all negative.

Taking into consideration of obese physique of the patient, to minimize the corticosteroid dosage to the greatest extent possible, we adopted a treatment regimen of 30 miligram(mg) prednisone together with two 1 gram(g) infusions of rituximab at 2-weeks interval. One month after the second infusion of rituximab, the patient had her first outpatient follow-up visit. It was found that the 24-hour urine protein had decreased from the peak value of 8.8g to 2.2g, and the albumin level had risen from the lowest 1.7g/dl during hospitalization to 2.5g/dl.Then we administered the SGLT2 inhibitor dapagliflozin to the patient, but after one week of use, the treatment was discontinued due to a urinary tract infection. In the meantime, the angiotensin II blocker irbesartan was gradually increased to double the dose within one month while maintaining the patient’s blood pressure tolerance. The dose of glucocorticoid should be gradully tapered off in accordance with the doctor’s instructions, with a monthly reduction of 5 milligram until the medication was discontinued. During outpatient follow-up, the patient’s 24-hour urinary protein level remained around 2g, consequently, an additional 1g of rituximab was administered after half a year, that is, in January 2023. Thereafter, the 24-hour protein dropped to 1.3g. Unfortunately, the patient was lost to follow-up after this.

## Discussion

In this case, we present a 48-year-old female patient with pSS who developed membranous nephropathy as an extraglandular manifestation. Primary Sjögren’s syndrome is a chronic and autoimmune disorder, targeting the exocrine glands especially the lacrimal and salivary glands, with the consequence of dry eyes and dry mouth (13). However, the inflammatory process in pSS extends beyond the exocrine glands, involving multiple organs including the kidney. Renal involvement occurs in approximately 5% of patients with pSS, manifesting primarily as tubulointerstitial nephritis due to lymphocytic infiltration or, less commonly, as immune complex-related glomerulonephritis (6). In our patient, renal biopsy revealed features consistent with membranous nephropathy secondary to pSS, including widespread mesangial hypercellularity and mesangial matrix expansion, thickening of the glomerular basement membrane with prominent spikes, and diffuse fine granular deposits of IgG along the capillary loops and mesangial areas under immunofluorescence microscopy. These findings highlight the complexity of renal manifestations in pSS and underscore the importance of comprehensive evaluation for accurate diagnosis and appropriate management.

Sjögren’s syndrome can occur in isolation or in a secondary form alongside with other autoimmune disorders such as scleroderma, rheumatoid arthritis and systematic lupus erythematosus (14, 15). This patient has the symptoms of dry eyes and dry mouth with positive results of ANA, Anti-SS-A/Ro antibody and ANCA, whether it is primary or secondary to SLE or ANCA?

pSS is more likely to occur in women in a female-to-male ratio of 9, with an incidence of 0.3%-0.5% in the population aged 40 years or older (16). Currently, the commonly uesd diagnostic criterion for Sjögren’s syndrome is the 2016 American College of Rheumatology(ACR)/European League Against Rheumatism(EULAR) classification, which is a robust criteria in accordance with a combination of laboratory results and clinical features aggregated weighted score of five objectives (17, 18): Schirmer’s test(<5mm in 5 minutes), lissamine green or fluorescein staining(≥4 in Van Bijsterveld Score or ≥5 in Ocular Staining Score) and unstimulated salivary flow rate(≤ 0.1mL/minute), each scoring 1 point; anti-Ro/SSA positivity and lip biopsy presented focal lymphocytic sialadenitis with number of foci ≥ 50 lymphocytes per 4 mm^2^ of tissue, each scoring 3 points. A total score of ≥4 points with either oral or ocular dryness is diagnostic of SS. To sum up, the patient meets the diagnostic criteria based on those that oral and ocular dryness, positive antibody, unstimulated salivary flow rat, tear break-up time as well as lip biopsy results. Furthermore, the patient does not exhibit lupus symptoms as well as the antibodies related to lupus were all negtive. The patient does not meet the diagnostic criteria for systemic lupus erythematosus (SLE) as outlined by either the 2022 Systemic Lupus International Collaborating Clinics (SLICC) classification criteria or the 2019 EULAR/ACR classification criteria.

Anti-neutrophil cytoplasmic antibody (ANCA) - associated vasculitis(AAV) has been described in pSS with a pervasiveness in approximately 6-7% patients, most commonly p-ANCA associated vasculitis with anti-MPO specificity (19). Nevertheless, ANCA positivity does not mean that the existence of an underlying ANCA-associated vasculitis (20). AAV onset seems to be related with pSS extraglandular manifestations.The pathogenesis of AAV in pSS may involve shared pathways, such as B-cell dysregulation and autoantibody production. Hypergammaglobulinemia and cryoglobulinemia, common in pSS, may exacerbate immune-complex deposition, while ANCA (e.g., anti-MPO) directly targets vascular endothelia, amplifying inflammation (19, 21).

Kidney is one of the important extraglandular organs involved in primary Sjogren’s syndrome, lying in two underlying physiopathologic mechanism: epithelial disease primarily characterized by mononuclear lymphocyte infiltration, leading to tubulointerstitial nephritis, and non-epithelial diseases accompanied by secondary immune complex-mediated glomerular lesions (22). Glomerular involvement in pSS is far less usual than tubulointerstitium. According to literature reports that common glomerular diseases associated with pSS are membranous nephropathy, mesangioproliferative glomerulonephritis and membranoproliferative glomerulonephritis (7, 8). Approximately 75% cases with uncertain etiology are termed as primary membranous nephropathy which is not associated with any potentially causal and recognizable systemic disease, drug exposure, or infection, presenting as a renal-limited disease, as a result (23). Conversely, secondary membranous nephropathy is associated with a particular cause, such as autoimmune diseases, chronic infection, hematopoietic stem cell transplantation, neoplasia, sarcoidosis and drugs and/or toxins (24). So in our case, what on earth it is a distinct entity overlapping with pSS namely primary membranous nephropathy(PMN) or it is a unique subset of pSS-related renal involvement, scilicet secondary membranous nephropathy(SMN)? To our knowledge, it is difficult to identify whether it is primary or secondary membranous nephropathy, which usually requires comprehensive judgments based on clinical features, serological detection and pathology. Male gender distribution is prevalent in PMN as opposed to female predominance in SMN (25). Comorbidities, such as hypertension, edema, infection, diabetes, embolism, and nephrotic syndrome, are comparable in PMN and SMN cases, excluded from microscopic hematuria, which is substantially linked to SMN (25). Almost 85% of primary membranous nephropathy is mediated by antibodies targeting the M-type phospholipase A2 receptor(anti-PLA2R), exceeding 90% specificity and 70% sensitivity, while thrombospondin type 1 domain-containing 7A(THSD7A)is involved in 3%-5% cases (25, 26). In addition, auto-antibodies to both PLA2R and THSD7A are predominantly of the IgG4 subclass and both of which the two proteins are highly expressed in podocytes than in glomerular mesangial and endothelial cells (27, 28).All patients are diagnosed on the basis of diffuse and global GBM thickening on light microscopy, with Silver Methanamine stain revealing prominent spikes along the GBM (25). By contrast, predominant fine granular positive C3c and IgG(especially IgG4) colocalized with PLA2R or THSD7A along the GBM in a case of nephrotic syndrome indicates PMN, while SMN is diagnosed when a patient exhibits nephritic syndrome along with the presence of endocapillary hypercellularity, mesangial cell proliferation, and expansion of the mesangial matrix under light microscopy (LM), as well as a heightened incidence of IgA, C3c, and C1q detected by direct immunofluorescence (DIF) microscopy.For a definitive diagnosis, electron microscopy is necessary, as it reveals the presence of exclusive subepithelial electron-dense deposits (EDD) in cases of Primary Membranous Nephropathy (PMN), whereas Secondary Membranous Nephropathy (SMN) demonstrates subendothelial, mesangial, and intramembranous EDD (29–32). So go back to our patient, she is seronegative for both antigens, with fine granular deposition of IgG(mainly IgG1 and IgG2) along the capillary walls and in the mesangium under immunofluorescence microscopy as well as a large amount of electron-dense deposites observed in subepithelial, intramembranous and mesangial regions. IgA, C4c, IgG3, IgG4, PLA2R, THSD7A and fibrinogen were negative.Based on the above results, we are more inclined to diagnose secondary membranous nephropathy.

Neuromyelitis optica spectrum disorder is an autoimmune astrocytopathy characterized by patients presenting with one or more of six clinical manifestations and is related to anti-aquaporin antibody, which plays a crucial role in pathogenesis, diagnosis and prognosis, has the ability to penetrate the blood-brain barrier. This triggers an immune response, resulting in inflammatory cell infiltration, demyelinating lesiongs and hyaline degeneration of blood vessels (33–35). The possible mechanism underlying the coexistence of NMOSD and SS may lie in that AQP4 is lowly expressed in salivary and lacrimal glands contrast to the high expression of AQP5 associated the pathogenesis of SS (36).AQP4 and AQP5 share with 50% common genetic sequence resulting in anti-AQP4 antibody binding to both AQP4 and AQP5 in multiple sites such as brain and exocrine glands. Additionally, anti-SSA antibodies interact with SSA-antigens, triggering immune-mediated inflammatory reactions to destroy the blood-brain-barrier thereby facilitating the entry of anti-AQP4 antibodies into the central nervous system (37, 38). A case report has described the coexistence of membranous nephropathy and neuromyelitis optica, but the relationsheep between them has not yet been definitively established (39).We boldly hypothesize that there may exist a specific mechanism that can trigger these three diseases stimultaneously, possible reasons including immunologic regulationg disorder, interaction of the multiple targets with autoantibodies, genetic predisposition, and shared common antigens which trigger a cross immune response, which need fuether research to confirm.

The treatment approaches of Sjögren’s syndrome is still limited to symptom-focused interventions and based on broad-soectrum immunosuppression for systemic disease in recent decades, with deficient information on the effectiveness and safety of the available treatment options (40). There is no single treatment regimen that can simultaneously address glandular symptoms and systemic organ complications. Rituximab for the treatment of Sjögren’s syndrome has mostly been studied in small samples, with mixed reports on its effectiveness, while it is recommended as a secondline or rescue therapy for those patients complicated by severe and refractory systemic disease such as glandular involvement, arthritis, cutaneous vasculitis, interstitial pneumonia, glomerulonephritis, multineuritis, CNS vasculitis, NMOSD as well as hemolytic anemia (41). Taking into account the patient’s overweight condition an well as concomitant membranous nephropathy, in order to minimize the dosage of glucocorticoids as much as possible, we choose the regimen of 30mg prednisone acetate combined with rituximab with a predominant use of 2 doses of 1 g each administered 15 days apart. As is well known, rituximab is a human/murine chimeric monoclonal antibody that specifically targeted against CD20 antigen resulting in B cell depletion. Therefore, peripheral B-cell depletion is indeed a well-established pharmacodynamic marker of rituximab activity. Unfortunately, B-cell count measurement is unavailable in our medical institution, which may weaken the mechanistic link between treatment and outcome. Although this patient was lost to follow-up, the current results indicate that the patient’s proteinuria has been significantly alleviated.

In summary, this case demonstrates the effectiveness of rituximab in managing secondary membranous nephropathy associated with primary Sjögren’s syndrome (pSS). The patient exhibited significant clinical improvement, as evidenced by a marked reduction in proteinuria levels following treatment with rituximab and corticosteroids. This highlights the potential role of B-cell depletion therapy in controlling both renal and systemic manifestations of pSS. However, distinguishing between primary and secondary membranous nephropathy remains challenging and necessitates comprehensive evaluations involving clinical features, serological markers, and pathological findings. Our case supports the notion that secondary membranous nephropathy is more likely when fine granular deposits of IgG (predominantly IgG1 and IgG2) and electron-dense deposits are observed in subepithelial, intramembranous, and mesangial regions. Furthermore, the coexistence of neuromyelitis optica spectrum disorder (NMOSD) and pSS may be explained by shared pathogenic mechanisms involving aquaporin-4 (AQP4) and AQP5 antibodies. Future studies are warranted to elucidate the underlying mechanisms and optimize treatment strategies for patients with pSS-associated membranous nephropathy, potentially paving the way for personalized therapeutic approaches.

## Data Availability

The raw data supporting the conclusions of this article will be made available by the authors, without undue reservation.
